# GENDER DISPARITIES IN THE MENTAL HEALTH OF CAREGIVERS BEFORE AND DURING THE COVID-19 PANDEMIC

**DOI:** 10.1093/geroni/igad104.1846

**Published:** 2023-12-21

**Authors:** Lucie Kalousova, Brina Ratangee

**Affiliations:** Vanderbilt University, Nashville, Tennessee, United States; Vanderbilt University, Nashville, Tennessee, United States

## Abstract

Prior research has shown that many have experienced worsened mental health during the COVID-19 pandemic. This study uses a large, nationally representative sample of caregivers interviewed by the Behavioral Risk Factor Surveillance System (BRFSS). It implements regression models to examine the changes in mental health among informal caregivers for older adults, who are a high-risk group for COVID-19 infection. We ask: (1) has the average number of poor mental health days among caregivers increased during the pandemic; (2) has the increase varied by gender; (3) the condition of the person receiving care? Our results indicate that caregivers experienced a sharp increase in poor mental health outcomes at the onset of the pandemic, much sharper than non-caregivers. In 2021 and 2022, male caregivers’ mental health returned to pre-pandemic levels, while women’s caregivers did not. We hypothesize that gender disparity in mental health outcomes could be attributed to differences in the types of caregiving responsibilities, the level of stress experienced, and the social support available. Furthermore, our study also shows that caregivers providing care to Alzheimer’s disease (AD) patients have worse mental health outcomes than other caregivers. Women who care for AD patients have worse mental health than male AD caregivers, whose mental health did not significantly differ from other caregivers. The findings could inform policy decisions and support the development of interventions that aim to mitigate the negative impact of the pandemic on caregivers’ mental health.

